# Nanoporous PbSe–SiO_2_ Thermoelectric Composites

**DOI:** 10.1002/advs.201700199

**Published:** 2017-08-11

**Authors:** Chao‐Feng Wu, Tian‐Ran Wei, Fu‐Hua Sun, Jing‐Feng Li

**Affiliations:** ^1^ State Key Laboratory of New Ceramics and Fine Processing School of Materials Science and Engineering Tsinghua University Beijing 100084 P. R. China

**Keywords:** mechanical alloying, nanoporous structures, thermoelectric composites

## Abstract

Nanoporous architecture has long been predicted theoretically for its proficiency in suppressing thermal conduction, but less concerned as a practical approach for better thermoelectric materials hitherto probably due to its technical challenges. This article demonstrates a study on nanoporous PbSe–SiO_2_ composites fabricated by a facile method of mechanical alloying assisted by subsequent wet‐milling and then spark plasma sintering. Owing to the formation of random nanopores and additional interface scattering, the lattice thermal conductivity is limited to a value as low as 0.56 W m^−1^ K^−1^ at above 600 K, almost the same low level achieved by introducing nanoscale precipitates. Besides, the room‐temperature electrical transport is found to be dominated by the grain‐boundary potential barrier scattering, whose effect fades away with increasing temperatures. Consequently, a maximum *ZT* of 1.15 at 823 K is achieved in the PbSe + 0.7 vol% SiO_2_ composition with >20% increase in average *ZT*, indicating the great potential of nanoporous structuring toward high thermoelectric conversion efficiency.

## 1 Introduction

With the advent of efficient materials, thermoelectric (TE) power generation technology is gradually evolving as a suitable solution to harvest wasted heat energy sources.^1^ The performance of a TE material is mainly judged by its dimensionless figure of merit (*ZT*) defined as *ZT* = *S* ^2^ *σT*/(κ_e_ + κ_L_), where *S*, σ, and *T* are the Seebeck coefficient, electrical conductivity, and absolute temperature, respectively, while κ_e_ and κ_L_ are the electronic and lattice contributions to the thermal conductivity. A powerful strategy towards higher *ZT* is to lower lattice thermal conductivity (κ_L_), in which various methods, such as nanostructural engineering,^2^, ^3^, ^4^, ^5^ alloying (introducing mass and size disorder),^6^, ^7^, ^8^ and constructing complex crystal structures^9^, ^10^ have been utilized.

For decades, nanoporous structure has been theoretically demonstrated effective in lowering thermal conductivity,^11^, ^12^, ^13^ which should be a promising routine toward high‐efficiency TE materials but has been more or less overlooked for a long time. Until recent years, increasing attention was paid on this strategy, for example, calculations by Prasher^13^ had suggested that diffuse scattering at medium/pore interfaces would significantly reduce the thermal conductivity of the medium. Afterwards, Romano and Grossman^14^ further identified the pore configuration that minimized thermal transport. Experimentally, by introducing hollow structures, Wu and co‐workers successfully reduced the thermal conductivity of Bi_2_Te_3_ to a low value of 0.5 W m^−1^ K^−1^.^15^ Compared with the well‐established nanostructuring techniques, such as introducing nano‐inclusions/precipitates^2^, ^3^ or forming nanocomposites^9^ with embedded nanoparticles, nanoporous structuring has the following advantages. First, nanopores have no influences on the chemical compositions of the matrix material. Second, nanopores are likely more stable at high temperatures than the precipitates that are usually formed by annealing. In addition, increasing pores can reduce the use of TE materials. However, related studies to date were limited to a few TE material systems,^16^, ^17^, ^18^, ^19^, ^20^ such as SiGe‐based alloys,^16^ bismuth tellurides^17^ and some oxides.^18^, ^19^ In the past decade, more studies have been devoted to searching for new TE materials and their chemical modifications for TE performance enhancement, while less attention has been paid to the importance of nanoporous structures. Only a few methods were reported on engineering the thermal transport of TE materials by introducing nanoporous structures, such as altering the sintering processes,^17^ employing pore‐forming agents,^21^ or using wet‐chemical synthesis to prepare hollow structured precursors.^15^, ^18^ This may also be due to the lack of techniques in precisely controlling the pore size, alignment, and configuration, etc.

In this paper, we reported a systematic study on nanoporous PbSe–SiO_2_ TE composites, in which nanosized SiO_2_ particles were used to create randomly distributed nanopores via a facile milling processing (mechanical alloying, MA), leading to greatly reduced thermal conductivity. In addition, the electrical transport was found to be governed by the grain‐boundary potential barrier scattering (for simplicity, using “boundary‐barrier scattering” instead in the following text) that is related to the incorporation of SiO_2_ nanoparticles and the presence of nanopores. A considerable *ZT* enhancement was achieved at high temperatures with the maximum value being 1.15 at 823 K, indicating the effectiveness of nanoporous structuring as a promising technique towards high TE performance.

## 2 Results and Discussion

X‐ray diffractions revealed no significant differences among the compositions of PbSe + *x* vol% SiO_2_, indicating no other than the PbSe matrix with *Fm* $\bar{3}$ *m* symmetry, as depicted in Figure S1 (Supporting Information). In addition, no peaks related to oxides (either SiO_2_ or PbO) were observed but some traces of Pb peaks that appeared at around 32° (2θ) due to excessive Pb added. Accordingly, added SiO_2_ seemingly exhibits good chemical inertness during the fabrication process, which should benefit the electrical performances of these composites.

**Figure 1:**
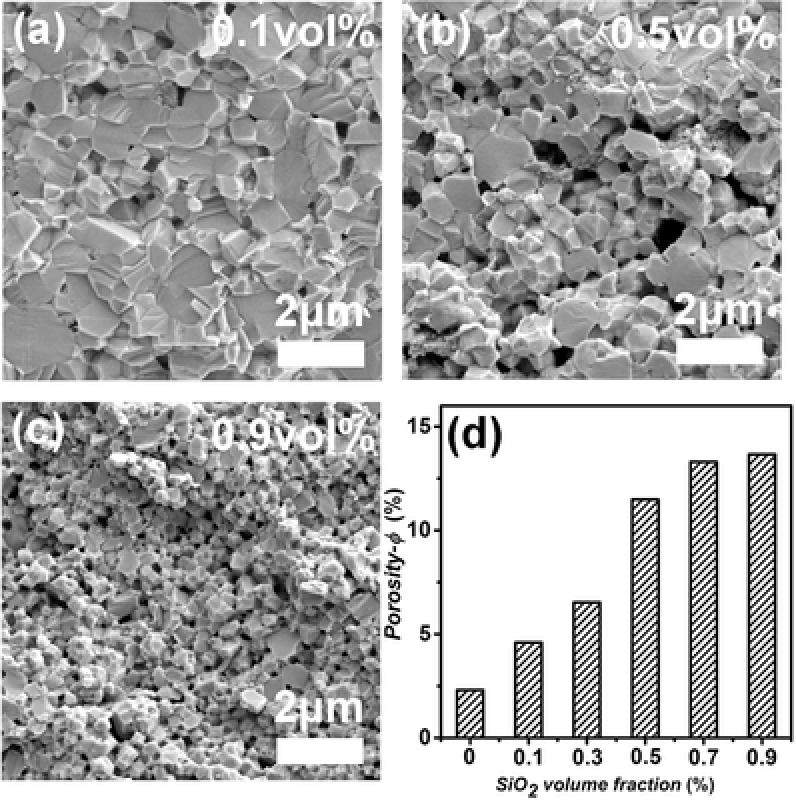
SEM morphologies for PbSe + *x* vol% SiO_2_ samples, a) *x* = 0.1, b) *x* = 0.5, c) *x* = 0.9; d) the calculated porosity for PbSe–SiO_2_ composites.

**Figure 2:**
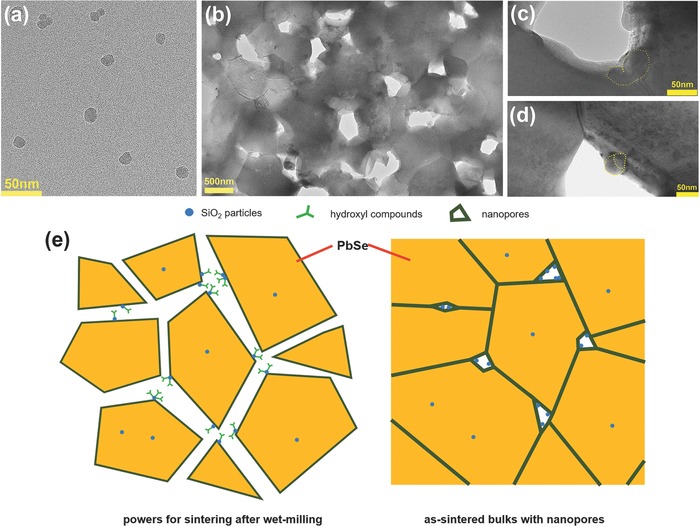
TEM morphologies for a) SiO_2_ particles, b) nanoporous structure of the 0.9 vol% sample, c,d) residual SiO_2_ particles around nanopores. e) A hypothesis for the formation of nanoporous structures in PbSe–SiO_2_ composites according to the TEM observations.

**Figure** **1** shows the micromorphologies of PbSe–SiO_2_ composites on fractured surfaces. At first sight, an obvious decrease in grain size was easily noticed with increasing SiO_2_ addition, that is, samples containing small amount (e.g., 0.1 vol%) of SiO_2_ were characterized with larger grains of 1–2 µm. On the contrast, for SiO_2_‐rich compositions like the 0.9 vol% one, most grains were refined to smaller dimensions ≈400 nm, suggesting a successful suppression on grain growth by adding nano‐SiO_2_ particles. Besides, compositing with SiO_2_ also brought about a remarkable increase in the porosity (φ = 1−ρ/ρ_0_, where ρ and ρ_0_ are the measured and theoretical densities) of almost all compositions, as listed in Figure 1d. For *x* = 0.9, a large porosity of 14% was found accompanied with prevalent nanopores throughout the matrix as depicted in Figure 1c and **Figure** **2**.

The nanopores are speculated to be induced by the SiO_2_ nanoparticles, probably ascribed to the good hydrophilia^22^ and abundant surfaces of nano‐SiO_2_ particles, where the absorbed hydroxyl compounds (water and ethanol from wet‐milling procedure) are vaporized during the sintering process and thus leave nanopores behind, as proposed in Figure 2. Intuitively, both refined grains and increased nanoporosity should be beneficial for scattering phonons with low‐to‐mediate frequencies so as to limit the lattice thermal conductivity, which will be shown later.

**Figure 3:**
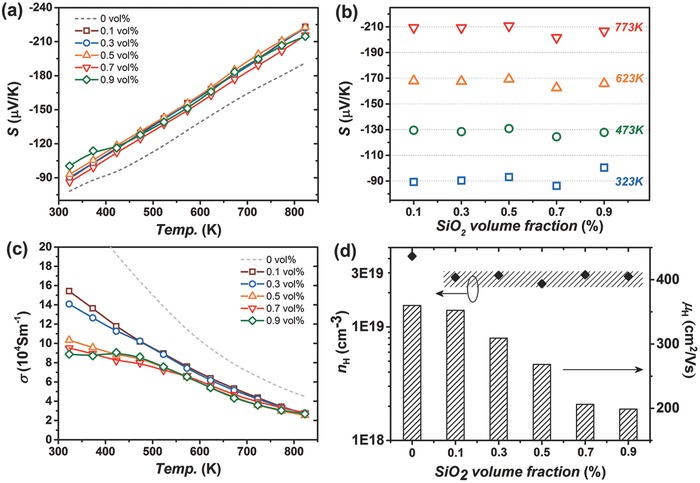
Seebeck coefficients as a function of a) temperature and b) SiO_2_ content. c) Electrical conductivity values, where the dashed gray line refers to data of SiO_2_‐free PbSe_0.998_Cl_0.02_ in our previous work. d) Hall carrier concentration and mobility of PbSe–SiO_2_ composites with various SiO_2_ loads at 323 K.

All compositions exhibited typically degenerate behaviors, as the Seebeck coefficients increased linearly against temperature as shown in **Figure** **3**. Since a similar doping level (0.2 mol% Cl) was intentionally controlled, different samples exhibited quite close thermopower values especially at elevated temperatures, although being slightly larger than that of the SiO_2_‐free one probably due to additional scattering and lower carrier concentration as shown latter in Figure 3d. Indeed, at lower temperatures, the carrier transport seems more sensitive to other scattering centers apart from the acoustic phonons, for example, grain boundaries and nanopores, which leads to appreciable deviation from the common negative correlation between Seebeck coefficients and carrier concentration.^23^

Despite the small differences in thermopower, the electrical conductivity varied a lot among different compositions. An obvious decrease in electrical conductivity was observed with increasing SiO_2_ content in Figure 3c. Particularly, for samples with SiO_2_ contents over 0.5 vol%, especially the 0.9 vol% one, the electrical conductivity deviated from the normally decreasing tendency near room‐temperature (RT), showing a “bump” between 323 and 523 K. Such an “abnormal” trend is ascribed to additional scattering processes, probably by grain boundaries or nanopores that involve energy barriers and exhibit different temperature dependence from the dominant acoustic phonon scattering. Nevertheless, as the temperature increases, free carriers become more energetic so that they experience less impact from energy‐independent scattering centers like nanopores, causing limited damages to the high‐temperature TE performances.

Figure 3d shows Hall carrier concentration and mobility at 323 K as functions of SiO_2_ volume fraction, where all SiO_2_‐containing compositions exhibited similar *n*_H_ levels, but smaller than that of the SiO_2_‐free one, which is probably ascribed to the element Cl loss during the synthesis with the existence of SiO_2_ particles. Besides, carriers from different samples distinguished themselves from others by the quite different mobility (*µ*_H_) values. As can be seen, the *µ*_H_ values were less changed with only 0.1 vol% SiO_2_ loaded, persisting ≈350 cm^2^ V^−1^ s^−1^ but straightly downward to ≈200 cm^2^ V^−1^ s^−1^ when the SiO_2_ content was up to 0.9 vol%.

**Figure 4:**
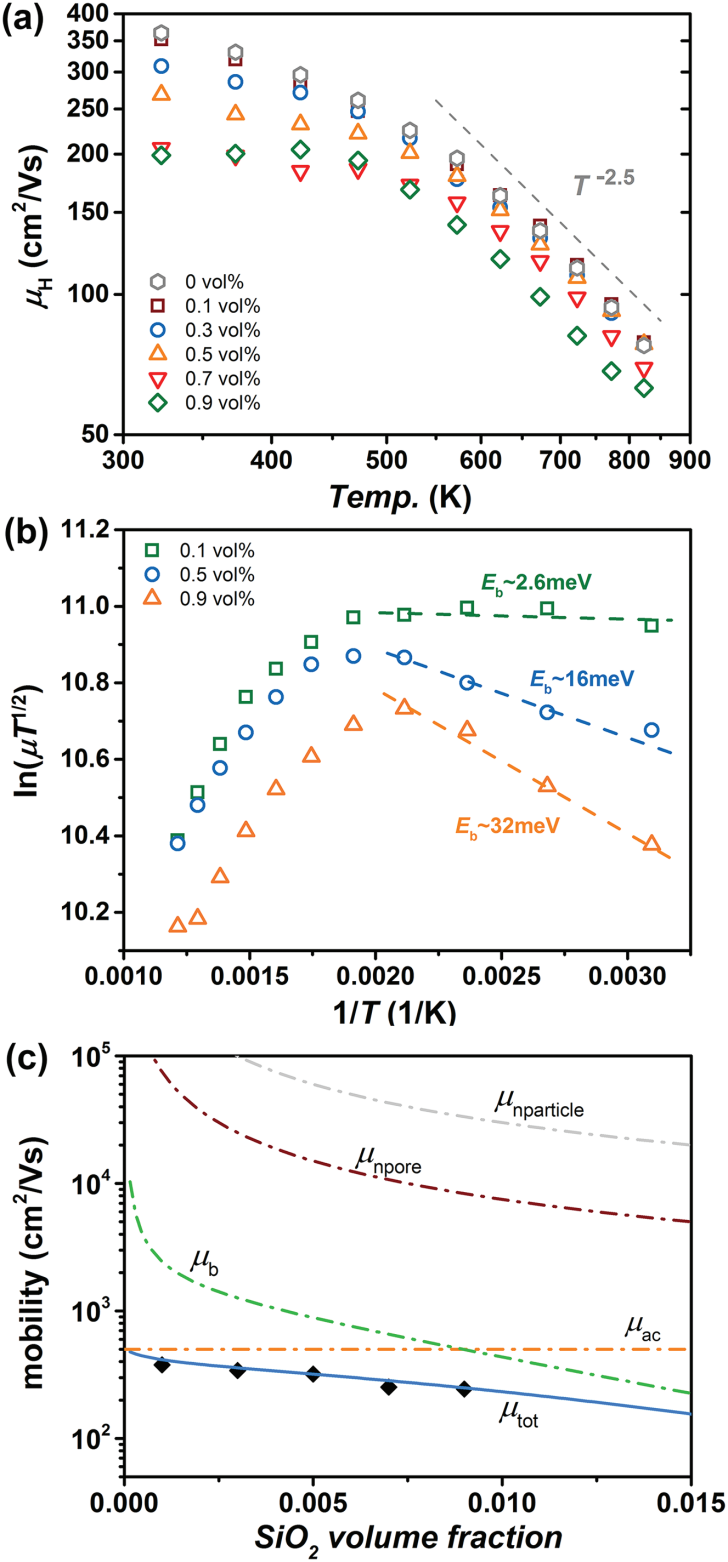
a) Hall mobility values as a function of temperature, and b) energy barrier estimation by fitting Ln(*µT* ^1/2^) vs. 1/*T*, where the boundary barriers were roughly derived to be 2.6, 16, and 32 meV for 0.1, 0.5, and 0.9 vol% samples, respectively. c) The construction of the total mobility (with the composite effect extracted) from different components, including acoustic phonon scattering, boundary‐barrier scattering and nanoparticle/pore scattering as a function of *x*.

Such a drop of mobility could be naturally ascribed to the incorporated SiO_2_ nanoparticles and resultant nanoporous structures, that is, nanoparticle scattering, nanopore scattering, composite effect, SiO_2_/matrix and pore/matrix interface scattering. Actually, nanoparticle scattering (as well as nanopore scattering) can be easily estimated by a typical nanoparticle scattering model^24^

(1) $$\mu_{\mathrm{np}}=\frac{eV}{\sigma_{e}v_{e}m_{I}^{*}}$$

where *V* is the average volume of an individual nanoparticle/nanopore, σ_e_ the scattering cross‐section and *v*_e_ the electron velocity. As calculated in **Figure** **4**c, the contributions from these parts (*µ*_nparticle_ and *µ*_npore_) are relatively small, indicating that secondary SiO_2_ particles themselves, as well as nanopores, seemingly play a minor role here in scattering electrons mainly due to their small amounts (corresponding to a bigger “*V*”).

In addition, the large porosity can generate more immediate impacts upon the carriers, namely the composite effect, as seen from the similar dependence of φ and *µ* on *x* (Figures 1d and 3d), which can be easily quantified by the effective medium theories.^25^ Moreover, interface scattering (including particle/matrix and pore/matrix interfaces) is hard to be solely estimated here, for which it is taken as a part of another scattering mechanism discussed latter. At present, only the composite effect from the large porosity seems to account for the impaired mobility, however, as seen in Figure 4a, the temperature dependence of mobility is quite different for various compositions, indicating additional scattering mechanisms that cannot be simply ascribed to the composite effect. Indeed, polar optical scattering^26^, ^27^ (*µ*_po_ ≈ *T^−^* ^0.5^) should be partly responsible for the deviation from acoustic domination (*µ*_ac_ ≈ *T* ^−2.5^ for PbSe) here, but cannot explain the ascent in *µ* for *x* > 0.5. Instead, ionized impurity scattering, from which carriers experience scatterings with even a positive temperature dependence of *µ*_ii_ ≈ *T* ^1.5^, however, should also be excluded due to the large static dielectric constants for lead chalcogenides and the low impurity levels^27^ present in this work.

Instead, considering the nonexponential variation of *µ* and the presence of nanoporous structures, an additional scattering from boundary barriers is supposed to conform here, as previously observed in a variety of TE materials, for example, PbTe composites^28^ and SnSe polycrystals.^29^, ^30^ The boundary‐barrier scattering is considered as the carrier trapping at grain boundaries with thermally activated dynamics. The effective mobility under boundary‐barrier scattering is given by^31^

(2) $$\mu_{b}=\frac{Lq}{{\left(2\pi m*k_{B}T\right)}^{1/2}}e^{-\frac{E_{b}}{k_{B}T}}$$

where *L* is the effective grain size, *q* is the carrier charge, and *E*_b_ is the height of energy barrier in the depletion region. At lower temperatures, individual discrepancy in electron transport is observed especially for the 0.7 and 0.9 vol% samples, which is regarded as an effect from boundary‐barrier scattering induced by SiO_2_ addition. Figure 4b displays the scattering mechanism analysis upon the boundary‐barrier scattering model expressed as Equation (2). A good linearity of Ln (*µT* ^1/2^) against 1/*T* is obtained for each composition, with roughly estimated energy barriers (*E*_b_) of 32, 16, and 2.6 meV for the 0.9, 0.5, and 0.1 vol% samples respectively. The good linearity between these data further validates the existence of boundary‐barrier scattering in such PbSe–SiO_2_ composites.

Moreover, these energy barriers are believed to be generated by combined effects from refined grains and abundant nanopores generated by SiO_2_ incorporation, including the interface scattering from particle/matrix and pore/matrix interfaces. In fact, by a repeated MA‐SPS processing for the as‐sintered 0.9 vol% sample, the porous structure vanishes (Figure S4, Supporting Information) with the mass density increased to over 95%. In this procedure, the as‐sintered bulks were crushed and pulverized to powders again, which were subjected to secondary SPS. Different from the first one, there were less hydroxyl compounds absorbed to the SiO_2_ particles now, so less pores were created in the second sintering processing. As a result, the electrical performance was improved with decreased porosity as shown in **Table** **1** below, indicating the important role of nanopores instead of SiO_2_ themselves in dominating the electrical transport.

**Table 1:** TE parameters of the 0.9 vol% sample measured before (OM) and after repeated MA‐SPS (RM) processing

	*d* [g cm^−3^]	Porosity [φ]	ρ_323 K_ [mΩ cm]	*S*_323 K_ [µV K^−1^]	κ_L,773 K_ [W m^−1^ K^−1^]
OM	7.1	14.1%	1.12	−100	0.6
RM	7.9	4.5%	0.63	−88	0.7

Taking into account the composite effect, boundary‐barrier scattering and predominant acoustic phonon scattering (including polar optical scattering here), a good fitting between the mobility and SiO_2_ content is obtained as shown in Figure 4c, and these mobility components are also individually plotted, where the contribution from composite effect has been excluded beforehand. As is depicted, both scatterings from SiO_2_ particles and nanopores are negligible if compared with other mechanisms, such as acoustic phonons and boundary barriers, as we discussed above. Calculation details for mobility can be found in Supporting Information.

In addition, the boundary barrier is known as a thermally activated defect characterized with energy *E*_b_, so its influence is fading away with increasing temperature. Others, such as the composite effect, would retain its influence but with a smaller contribution (to the total mobility values) because of enhanced acoustic phonon scattering at higher temperatures. As shown in Figure 4a, the mobility (above 700 K) differentiates less at high temperatures among samples with a slight decrease upon increasing SiO_2_ content and obeyed typical *T* ^−2.5^ law for lead chalcogenides,^27^ suggesting the dominant role of acoustic phonon scattering on carriers. Thanks to limited influence from boundary‐barrier scattering, the high‐temperature electrical performance was less impaired by the SiO_2_ addition, providing certain possibility toward higher TE efficiencies.

**Figure 5:**
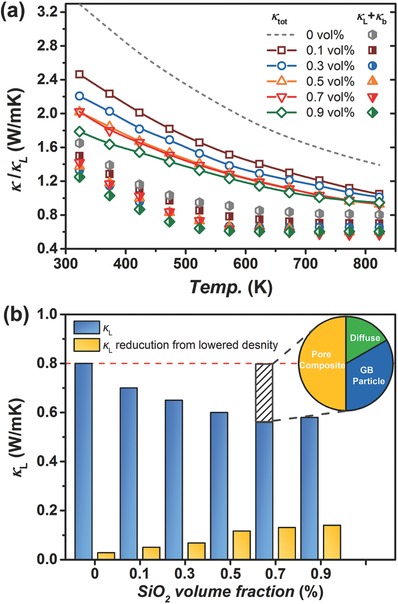
a) Total and lattice thermal conductivity values as a function of temperature, where the dashed gray line represents data of PbSe_0.998_Cl_0.002_ from our previous work. b) The κ_L_ reduction evaluation for nanoporous PbSe–SiO_2_ composites at 773 K, where the inset pie chart reveals the individual contribution of various scattering centers (including the pore composite effect, grain boundary and nanoparticles, and diffuse scattering) in reducing κ_L_ for the 0.7 vol% composition.

Apart from the impaired carrier mobility, phonons in PbSe matrix also experiences intensified scattering over a wide temperature range. **Figure** **5** shows the total (κ) and lattice (κ_L_) thermal conductivity values, where a declining tendency in both parameters was observed with increasing SiO_2_ content. Herein κ_L_ is roughly calculated by extracting the electronic part according to κ_e_ = *LσT* under the assumption of acoustic‐phonon scattering. Around RT, the κ_L_ decreases from 1.6 W m^−1^ K^−1^ to an average value of 1.3 W m^−1^ K^−1^ as SiO_2_ was incorporated. As temperature increases, κ_L_ decreases very quickly, downward to a plateau around 0.6 W m^−1^ K^−1^ before 600 K for most SiO_2_‐contained samples, which is much smaller than the average κ_L_ value of 0.8 W m^−1^ K^−1^ at 700–800 K for PbSe bulks.^32^, ^33^ The sharp decrease and the lower κ_L_ values are considered associated with the SiO_2_ addition, probably with multiple effects from the abundant nanopores, interfaces, refined grains and SiO_2_ nanoparticles.

As is known, at high temperatures phonons are more energetic with higher frequencies and smaller MFPs (several nanometers^34^ for PbSe), in which boundary scattering usually becomes so weak, especially for those with large grains. In contrast, nanopores often possesses smaller dimensions than grains, providing more possibilities for enhancing phonon scattering at higher temperatures, which is similar with the nanoparticle scattering.^26^ Additionally, for nanoporous structures, the existence of diffuse scattering^13^ from medium/pore boundaries would further contribute to the phonon scattering due to the surface roughness, which enlarges the potential of nanopores on reducing κ_L_ over a wider temperature region.

In order to assess the κ_L_ reduction via various scattering processes, we compared the variation of κ_L_ with both SiO_2_ content and porosity, and more close correlations are found between the κ_L_ reduction and the porosity, rather than the SiO_2_ content. Figure 5b displays the κ_L_ values as a function of SiO_2_ content at 773 K, where an obvious decrease is found with increasing SiO_2_ content up to 0.7 vol%, with κ_L_ values decreasing from 0.8 to 0.56 W m^−1^ K^−1^ almost linearly. Further addition of SiO_2_ seemingly causes less depression on phonon transport as the κ_L_ value does not decrease any more. The minimum κ_L_ was achieved in the 0.7 vol% sample, which is 30% lower than the average bulk values of 0.8 W m^−1^ K^−1^ and quite close to the limit obtained in nanostructured PbSe materials.^35^ This reveals great potential of nanoporous structuring as an effective approach for lowering lattice thermal conductivity. Besides, it should be mentioned that increasing porosity has another advantage of reducing the weight of the thermoelectric materials and hence the devices.^15^

As is stated before, the κ_L_ reduction here can be originated from various scattering centers, for example, nanoporous structures, boundaries, interfaces, or even SiO_2_ nanoparticles. The effective thermal conductivity of the nanoporous composites from the Maxwell–Garnett result is given by^36^

(3) $$\kappa_{\mathrm{eff}}=\left(1-\frac{1-\phi }{1+\frac{1}{2}\phi }\right)\kappa_{m}$$

where κ_eff_ is the effective conductivity of the nanoporous composites, φ is the porosity, and κ_m_ is the effective conductivity of the host medium. By substituting Equation (3) to the lattice thermal conductivity values, the effects on reducing thermal conductivity by nanoporous structuring can be more comprehensively understood. As depicted in Figure 5b (the yellow column and the inset), it is seen that the large porosity contributes a major part in help reducing the κ_L_, approximately 50% (0.13 of 0.24 W m^−1^ K^−1^) for the 0.7 vol% composition. In fact, as more SiO_2_ was added, the porosity was monotonously increased to the maximum value of 14% with SiO_2_ content up to 0.7 vol%, so did the κ_L_ reduction. Other possible scattering centers, like embedded SiO_2_ nanoparticles and grain boundaries, also work in limiting κ_L_ to some degree as indicated by the repeated MA‐SPS results (last column of Table 1). The diffuse scattering from pore/matrix interfaces, however, plays a minor role here with an estimation (Figure S6, Supporting Information) of 15%–25% contribution to the κ_L_ reduction (0.04–0.06 of 0.24 W m^−1^ K^−1^), probably due to the small MFPs of PbSe and large nanopore sizes.^12^

**Figure 6:**
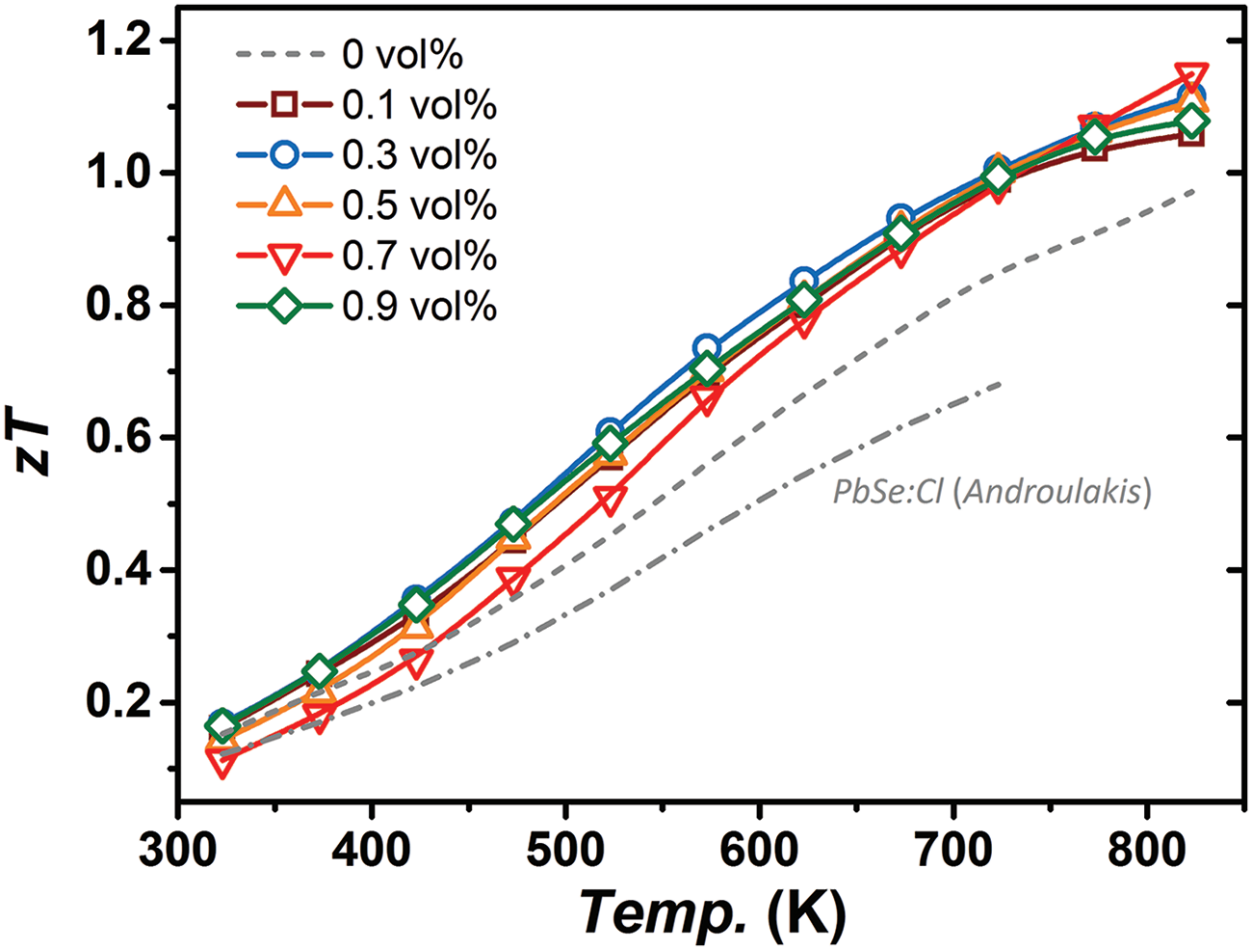
*ZT* values of PbSe + *x* vol% SiO_2_ samples in this work, with previous results on Cl‐doped PbSe as references.

As a result of the much lowered κ_L_ in nanoporous composites and less impaired electrical performance at high temperatures, a maximum *ZT* of 1.15 at 823 K was obtained in the 0.7 vol% sample, being an over 20% enhancement from n‐type PbSe with only Cl doped and nearly 40% from PbSe_1−_ *_x_*Cl*_x_* materials synthesized by melting techniques,^37^ as shown in **Figure** **6**. Noticeably, an obvious *ZT* enhancement around 600 K was observed at the same time, originating from the sharp decrease in thermal conductivity and the broad plateau of κ_L_ on the minimum level, contributing to the enhancement of average *ZT* at mediate temperatures. Actually, the average *ZT* between 523 and 823 K exceeds 20% improvement, increasing from 0.7 in the 0 vol% sample upwards to 0.9 in SiO_2_‐containing ones. These results suggested the feasibility and promise of nanoporous structuring as an effective method to modify transport properties and improve the performances of TE materials.

## 3 Conclusion

In summary, SiO_2_ addition has created abundant nanopores in PbSe–SiO_2_ composites by a facile powder‐based process. The existence of nanopores was found to introduce boundary barriers and impair charge transport but mainly at lower temperatures. Meanwhile, the porous structure also introduced additional scattering on phonons, thus significantly reducing the lattice thermal conductivity over a wide temperature range, leading to a maximum *ZT* of 1.15 in the composition of PbSe + 0.7 vol% SiO_2_ and also a higher average *ZT* at mediate temperatures. This work experimentally substantiated the great potential of nanoporous structuring as an effective approach toward high‐efficiency thermoelectrics.

## 4 Experimental Section

*Synthesis*: Bulk samples were synthesized by combining mechanical alloying (MA) and spark plasma sintering (SPS) using elemental Pb shots (99.999%, 1–3 mm), Se powders (99.99%, ≈75 µm), PbCl_2_ (99.9%, ≈75 µm) and nanosized SiO_2_ (99%, ≈10 nm) in the compositions of PbSe_0.998_Cl_0.002_ + *x* vol% SiO_2_ (*x* = 0, 0.1, 0.3, 0.5, 0.7, 0.9). The mixture of these powders was subjected to high‐energy ball milling within a stainless steel vessel filled with protective Argon gas. After milling at 450 rpm for 24 h, the MAed powders were washed down by a wet‐milling process using absolute ethanol for another hour. Afterward, the derived powders were vacuum‐dried at 310 K and then sintered into plates by SPS (SPS211Lx, Fuji Electronic, Japan) at 873 K for 5 min under an axial pressure of 50 MPa.

*Microscopic Identification*: The phase structure and microstructure of sintered samples were investigated by X‐ray diffractions using Cu‐K radiation (XRD, D8 Advance A25, Bruker, Germany), field emission scanning electron microscopy (FESEM, Merlin VP compact, Zeiss, Germany) and transmission electron microscopy (TEM, 2100, JEOL, Japan), respectively. The elemental distribution was also validated using electron probe microanalysis (EPMA, JXA‐8230, JEOL, Japan) on polished samples.

*Thermoelectric Measurement*: The Seebeck coefficient and electrical conductivity were measured by a Seebeck coefficient/electrical resistivity measuring system (ZEM‐2, Ulvac‐Riko, Japan) using bar samples, meanwhile, disk samples were machined for the thermal diffusivity (λ) measurements through the laser flash technique (TC9000, Ulvac‐Riko, Japan), where the thermal conductivity was derived from the formula κ = *λC*_p_ *d*, in which *C*_p_ is the heat capacity determined by the composite effect of PbSe and SiO_2_, and *d* is the mass density obtained by the Archimedes method. To identify electrical transport mechanisms, Hall measurements were conducted through the Van der Pauw technique under a reversible magnetic field of 0.52 T (8340DC, Toyo, Japan). The uncertainty of each parameter is estimated to be about 4%, which combined leads to an uncertainty of ≈15% in *ZT*.

## Conflict of Interest

The authors declare no conflict of interest.

## Supporting information

Supplementary
