# Supplementary material for: Nanoporous PbSe–SiO2 Thermoelectric Composites
Source: Adv Sci (Weinh). 2017 Aug 11;4(11):1700199. doi: 10.1002/advs.201700199 (PMC5700627; doi:10.1002/advs.201700199)
Supplement: Supplementary file 1 — Supplementary [file ADVS-4-na-s001.pdf]

Copyright WILEY-VCH Verlag GmbH & Co. KGaA, 69469 Weinheim, Germany, 2017.

ADVANCED SCIENCE
Open Access

Supporting Information

for *Adv. Sci.,* DOI: 10.1002/advs.201700199

Nanoporous PbSe–SiO_2 Thermoelectric Composites

*Chao-Feng Wu, Tian-Ran Wei, Fu-Hua Sun, and Jing-Feng Li**

Copyright WILEY-VCH Verlag GmbH & Co. KGaA, 69469 Weinheim, Germany, 2016.

Supporting Information

Nanoporous PbSe-SiO_2 thermoelectric composites

Chao-Feng Wu, Tian-Ran Wei and Jing-Feng Li*

I. XRD Results of PbSe + *x* vol% SiO_2 Bulks

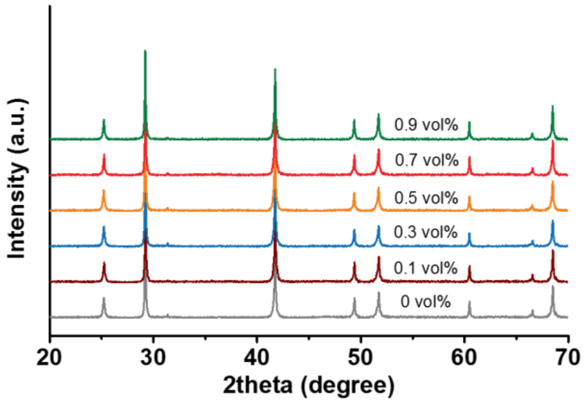

Figure S1. XRD patterns for PbSe + *x* vol % SiO_2 samples.

II. Electron Backscattered Images of 0.9 vol% sample

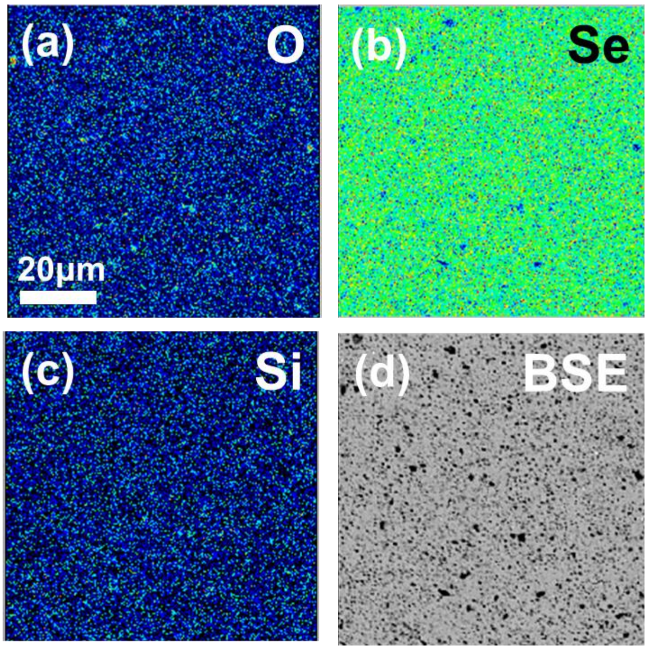

Figure S2. EMPA results of the 0.9 vol % sample, where the distribution of (a) O, (b) Se and (c) Si are show here. (d) BSE morphology of the same area.

III. Reproduction on the Electrical Performances of the 0.9 vol % sample

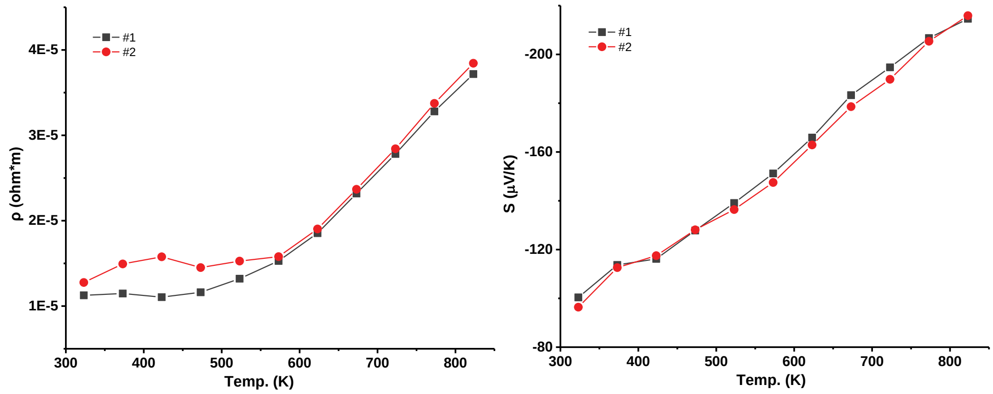

Figure S3. Reproducibility of the 0.9 vol % sample.

IV. SEM Morphologies of the 0.9 vol % sample before and after Repeated MA-SPS Processing

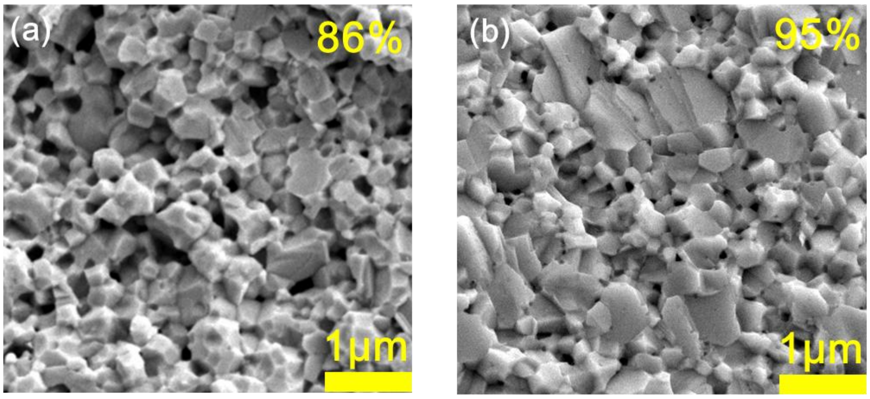

Figure S4. SEM of the 0.9 vol % sample: (a) before and (b) after repeated MA-SPS.

VI. Electrical Transport Analyzation on Mobility at near RT

Apart from the composite effects from porous structures, the carrier mobility at RT was supposed to be dominated by multiple mechanisms: acoustic phonons, boundary barriers and nanoparticle/nanopore scattering (characterized by a cross-section of scattering), thus the total mobility can be written in the form of the Matthiessen-type relations:

$$\frac{1}{\mu_{tot}} = \frac{1}{\mu_{ac}} + \frac{1}{\mu_b} + \frac{1}{\mu_{np}} \quad \text{(S1)}$$

where μ_{tot} is the total mobility for dense bulks derived from the MG theory,[1] μ_{ac} the individual mobility dominated by acoustic phonons (AC),[2] μ_b the individual mobility from boundary-barrier scattering (BB),[3] and μ_{np} the nanoparticle scattering (NP) dominant mobility,[4] respectively.

First, the mobility under acoustic phonon domination is expressed as in the framework of SKB model:

$$\mu_{ac} = \frac{2\pi\hbar^4 e C_l}{m_I^*(2m_b^* k_B T)^{3/2}\Xi^2} \frac{3\,{}^0F_{-2}^1}{{}^0F_0^{3/2}} \quad \text{(S2)}$$

Assuming little change in the elastic parameters by the SiO_2 addition and setting the temperature a constant, such as 323K, this mobility component would remain nearly constant.

Secondly, the carrier mobility dominated by boundary barrier scattering adopts the following expression:

$$\mu_b = Lq\left(\frac{1}{2\pi m^* k_B T}\right)^{1/2} exp\left(-\frac{E_b}{k_B T}\right) \quad \text{(S3)}$$

This barrier height was experimentally found quite relevant to the SiO_2 content, thus E_b here was simply derived from Figure 3(b) and Figure S4 as a function of the SiO_2 volume fraction χ (but actually the barrier height should be some combined effects as stated in the text, including the porosity ϕ to some degree; here we use the following relation for simplicity):

$$E_b \approx 3500\chi\ eV \quad \text{(S4)}$$

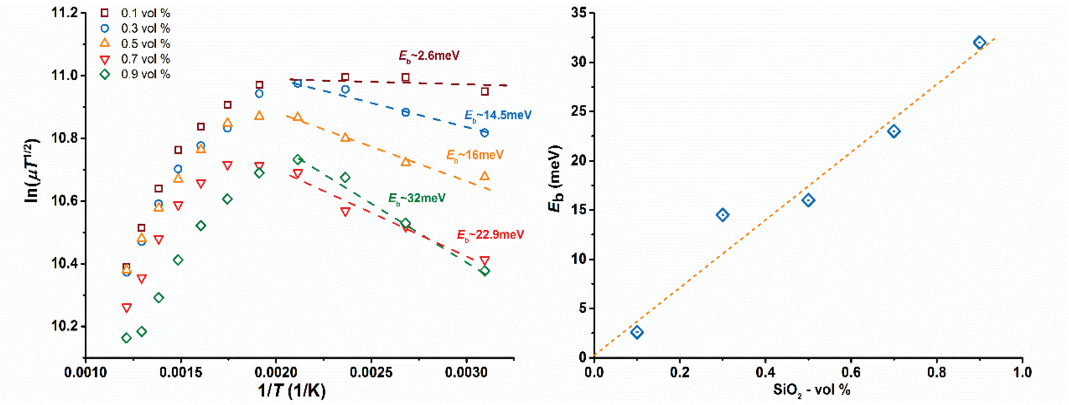

Figure S5. Energy barrier estimation on PbSe-SiO_2 composites.

Thirdly, the nanoparticle scattering (including nanopore scattering) is identified as:

$$\mu_{np} = \frac{eV}{\sigma_e v_e m_I^*} = \frac{eV_{np}}{\phi\sigma_e v_e m_I^*} \quad (for\ pore) \tag{S5}$$

$$or\ \frac{eV_{np}}{\chi\sigma_e v_e m_I^*} (for\ particle) \tag{S6}$$

where V_{np} is the average volume of an individual nanoparticle/nanopore. Taking v_e=10^5 m/s, roughly assuming $\phi = 15.5\chi$, and 20nm/15 nm for the average radius of nanopore/nanoparticle, respectively.

By subjecting these partitions to Equation S1, the total mobility (at 323K) could be roughly obtained as shown in Figure 3(c), where $\mu_{ac}^{323K} = 500\ \mathrm{cm^2V^{-1}s^{-1}}$ was reasonably suggested (this value is smaller than that predicted by Equation S2, as some underlying defects derived by MA processing also contribute the carrier scattering here, which is taken into the equivalent value of μ_{ac}). The resultant value of L, the effective distance of boundary barriers, was estimated to be 23(1+0.001/χ) nm, revealing a little smaller than the magnitude of grain sizes. However, it is perhaps reasonable sometime as the energy barriers are often related to the sub-grains or other crystal defects inside (especially in this work, the interface scattering from nanoparticles and nanopores), but not the real grain boundaries. The individual contribution from these scattering mechanisms can also be found in Figure 3(c), where both AC and BB mechanisms generated a

majority of impacts on the total mobility, however, the nanoparticle/nanopore played a tiny role here probably due to their small amounts and overlarge dimensions.

VII. Estimation of the κ_L reduction from diffuse scattering of nanopores

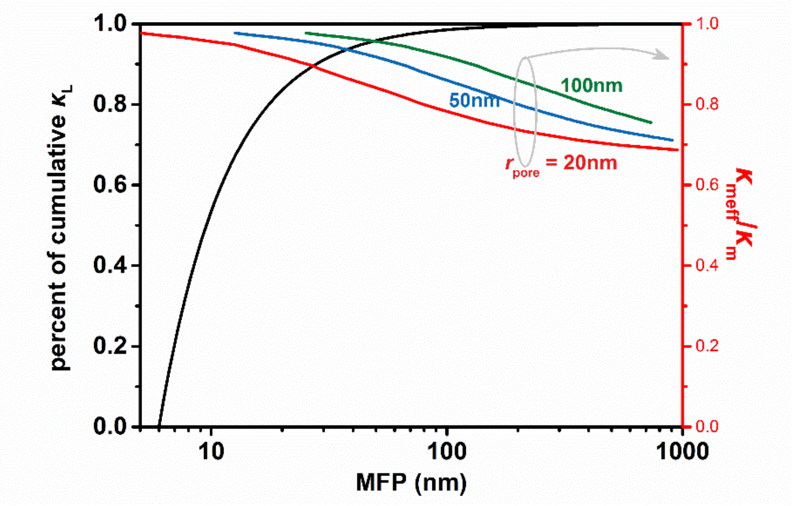

Figure S6. Estimation for the diffuse scattering on κ_L, where the left bar shows the accumulative κ_L calculated by the Debye model and acoustic phonon scattering, and the right bar was cited from ref.5 by assuming the pore radius of 20 nm, 50 nm and 100 nm, with the porosity of 15%. Here κ_{meff} refers to the modified κ_L of the matrix due to diffuse scattering from nanopores and κ_m the original κ_L value of the material. The average size (diameter) is determined to be 100~200 nm from the TEM images, thus the contribution of the diffuse scattering is estimated as 5-8% of the total κ_L (0.8W/mK), or 15-25% of the κ_L reduction (0.24 W m^{-1} K^{-1}) for the 0.7 vol % sample.

References

[1] Jamnik, J. R. Kalnin, E. A. Kotomin and J. Maier, *Phys. Chem. Chem. Phys.* **2006**, 8, 1310.

[2] H. Wang, Y. Z. Pei, A. D. LaLonde and G. J. Snyder, *P. Natl. Acad. Sci. USA*, **2012**, 109, 9705.

[3] J. Y. W. Seto, *J. Appl. Phys.* **1975**, 46, 5247.

[4] N. Mingo, D. Hauser, N. P. Kobayashi, M. Plissonnier and A. Shakouri, *Nano Lett.* **2009**, 9, 711.

[5] R. Prasher, *J. Appl. Phys.***2006**, 100, 34307.
